# The influence of the COVID‐19 pandemic on the spectrum of neonatal disease in a tertiary hospital in China: A retrospective analysis

**DOI:** 10.1002/hsr2.1928

**Published:** 2024-02-25

**Authors:** Yi Wang, Linhong Song, Ning Ma, Hao Li, Siqi Hu, Zhichun Feng

**Affiliations:** ^1^ Faculty of Pediatrics The Chinese PLA General Hospital Beijing China; ^2^ Institute of Pediatrics The Seventh Medical Center of PLA General Hospital Beijing China; ^3^ Beijing Key Laboratory of Pediatric Organ Failure Beijing China; ^4^ National Engineering Laboratory for Birth Defects Prevention and Control of Key Technology Beijing China; ^5^ Department of Pediatric Cardiac Surgery The Seventh Medical Center of Chinese PLA General Hospital Beijing China

**Keywords:** COVID‐19, disease spectrum, neonatal diseases, pediatrics

## Abstract

**Background and Aims:**

Neonatal diseases are a significant threat to global public health, affecting the homeostasis and well‐being of patients and reflecting the status of, and challenges to, regional, national, and global healthcare systems. This study sought to investigate how the disease spectrum observed among neonatal inpatients changed after the onset of the coronavirus disease 2019 (COVID‐19) pandemic.

**Methods:**

The present hospital‐based retrospective study analyzed the demographic and clinical characteristics of 19,943 hospitalized newborns from January 2018 to December 2022 using data derived from pediatric department registers.

**Results:**

According to the International Classification of Diseases 11th Revision (ICD‐11) classification criteria, the two most common neonatal disorders during this study period were “Certain conditions originating in the perinatal period” and “Disease of the respiratory system.” Following the start of the COVID‐19 pandemic (2020 onwards), the number of neonatal patients declined markedly (5742 per year vs. 2820 per year), and the incidence of “Disease of the respiratory system” was significantly lower than in 2018–2019 (25.72% vs. 17.46%).

**Conclusion:**

The study offers detailed insights into the shifts in neonatal disease patterns at the Seventh Medical Center of the PLA General Hospital following the onset of the COVID‐19 pandemic, providing a foundation for future research and policymaking efforts.

## Key messages

The disease spectrum observed among neonatal inpatients has changed markedly after the onset of the coronavirus disease 2019 (COVID‐19) pandemic. The study also analyzed changes in the disease spectrum among neonatal inpatients before and after lifting COVID‐19 lockdown measures. The epidemiological characterization of neonatal diseases can enhance understanding of their pathophysiological basis and aid in identifying disease‐related risk factors among professionals. These findings offer a valuable foundation for formulating health policies.

## 1 BACKGROUND

Health is vital to the well‐being of newborns, and the first 28 days of life, referred to as the neonatal period, is particularly important in this regard. During this neonatal period, babies face the highest risk of complications following birth, and this is also the period during which congenital conditions and congenital disabilities may first be identified.^1^ Neonatal disorders disrupt the homeostasis of a newborn's tissues, organs, and systems.^2^ Neonatal diseases require close monitoring as fetuses may be affected by inflammation and intrauterine infections, and birth itself is a significant physiological event. The organs of neonates, still in development, are more susceptible to harm.^3^, ^4^

Overall, neonatal health can reflect regional, national, and global healthcare challenges and progress, making it a key priority worldwide.^5^ Analyzing the spectrum of diseases facing neonates and other age groups can provide an evidence‐based foundation for efforts to prevent disease, treat patients, manage hospitals, and formulate appropriate policies.^6^

The advent of the coronavirus disease 2019 (COVID‐19) pandemic caused by severe acute respiratory syndrome coronavirus‐2 (SARS‐CoV‐2), which rapidly spread throughout the world after first emerging in December 2019,^7^, ^8^ has had a profound and sweeping impact on healthcare systems and society as a whole. Efforts to slow the spread of disease led to the introduction of hygiene‐focused countermeasures, including social distancing, hand washing, mask‐wearing, and reductions in contact numbers.^9^ Many countries adopted shelter‐in‐place policies to curb the pandemic.^10^ We hypothesized that this global paradigm shift may similarly have resulted in changes in the spectrum of neonatal diseases treated in hospitals. Indeed, several prior reports have focused on shifts in the disease spectrum during and after the COVID‐19 pandemic regarding the incidence of respiratory syncytial virus (RSV) infection,^11^ mental health disorders,^12^ and ocular trauma.^10^ Alkharsah et al. demonstrated a twofold increase in RSV‐positive cases in 2021 compared to the average positive cases from previous years.^11^ Researchers found that COVID‐19 outbreaks and related nonpharmaceutical interventions (NPIs) may have reduced influenza in Southern and Northern China and the United States by 79.2%, 79.4%, and 67.2%.^13^ In addition, clinical laboratories in the United States indicated a 61% decrease in the number of specimens submitted for influenza testing after late February 2020.^14^ The present retrospective study focused on inpatients treated in the PLA General Hospital in Beijing, China, from January 2018 to December 2022. Our hospital, an important center for pediatric care, serves patients from North China and has a well‐established neonatology unit. Using medical records, a clinical database was established to examine the neonatal disease spectrum in China from 2018 to 2022 and identify any pandemic‐related changes in this spectrum.

## 2 METHODOLOGY

This is a retrospective review of prospectively collected data. The study was performed at the Seventh Medical Center of the PLA General Hospital. All inpatients from the Division of Neonatology from 2018 to 2022 were enrolled, and clinical and other data were collected from pediatric department registers. A total of 19,943 patients were included in this study.

Clinical data, including profiles and demographic data, were collected from the electronic medical records and pediatric department registers of the Seventh Medical Center of the PLA General Hospital.

Descriptive statistics, such as age, region, International Classification of Diseases (ICD) diagnostic codes, and symptoms, were presented as frequencies and percentages using GraphPad Prism version 7 for Windows (GraphPad). The *χ* ^2^ test was used to infer any differences between categorical variables. All statistical tests were two‐sided, and *p* values < 0.05 were considered statistically significant, *p* values < 0.05 were considered statistically significant. All statistical analyses were done using Statistical Package for the Social Sciences (SPSS) software version 20 (IBM Corp.).

## 3 RESULTS

### 3.1 General overview

**Figure 1:**
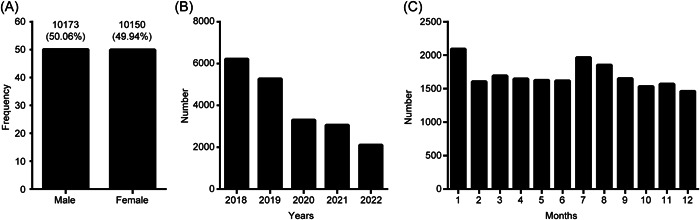
Neonatal disease distributions among inpatients in the Seventh Medical Center of the PLA General Hospital (2018–2022). (A) Gender distributions for inpatients. (B) Inpatient admissions by year. (C) Inpatient admissions by month.

In total, 19,943 inpatients were identified from 2018 to 2022 for an average of 3989 per year, of whom 49.94% and 50.06% were female and male, respectively (Figure 1A). Figure 1B shows a marked annual decline in the number of inpatients starting in 2020, coinciding with the onset of the COVID‐19 pandemic. Incidence distributions by month are shown in Figure 1C, revealing higher rates in January, July, and August.

### 3.2 Changes in disease distributions from 2018 to 2022

**Figure 2:**
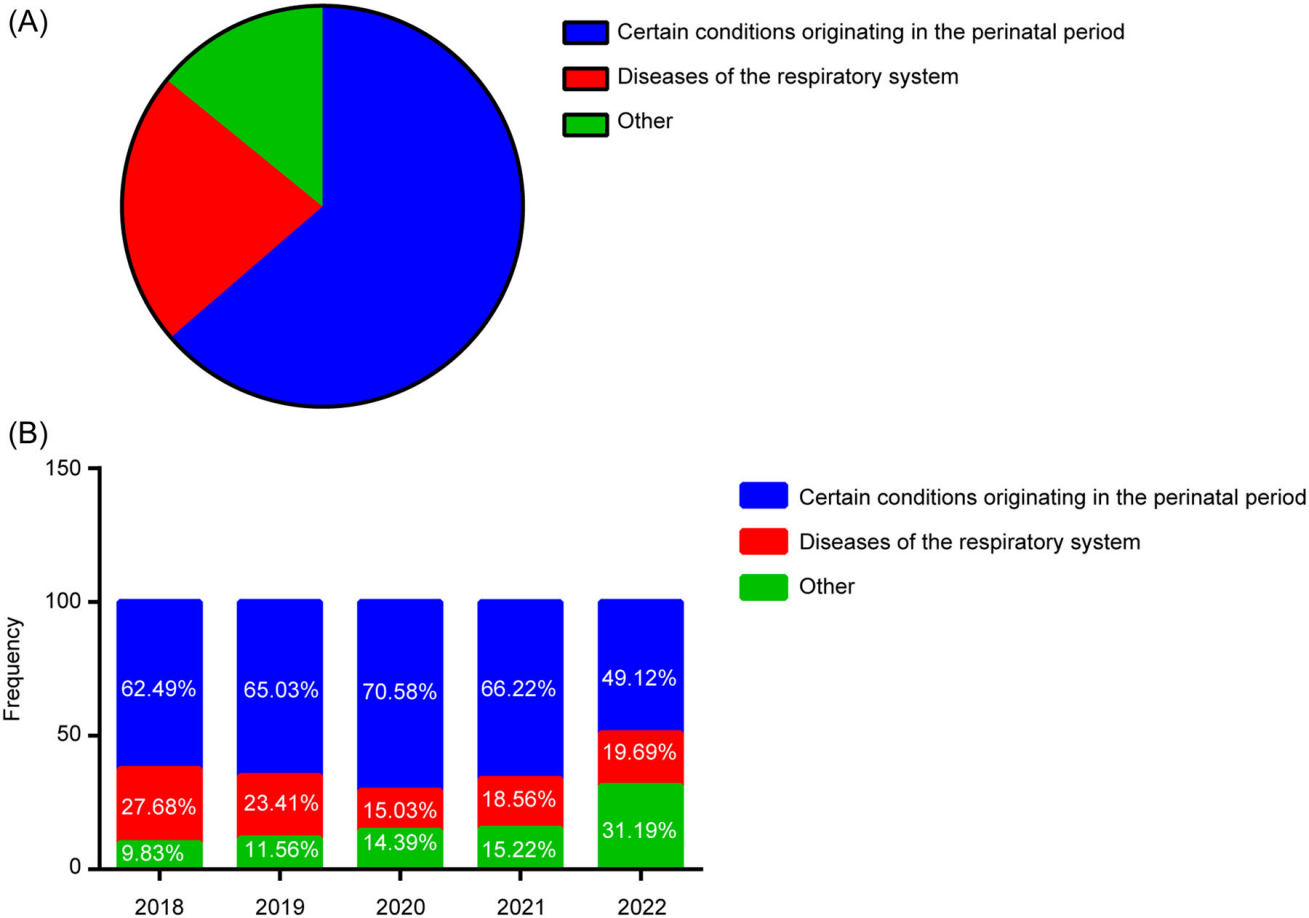
Disease Spectrum and frequencies among newborns in the Seventh Medical Center of the PLA General Hospital. (A) The disease spectrum and frequencies from 2018 to 2022. (B) The overall disease spectrum and frequencies during each year from 2018 to 2022.

The data were separated into two periods to assess the impact of the COVID‐19 pandemic on the spectrum of neonatal disease in our hospital. Period 1 (2018–2019) corresponds to the period before the outbreak of COVID‐19, whereas Period 2 (2020–2022) corresponds to the period following the start of the COVID‐19 pandemic. Based on ICD‐11 codes, 63.66% (*n* = 12,696) of the primary diagnoses were classified as “Certain conditions originating in the perinatal period.” “Disease of the respiratory system” was the second most common diagnosis (22.22%, *n* = 4431) (Figure 2A). During Period 1, 25.72% of children were diagnosed with “Disease of the respiratory system,” compared to 17.46% in Period 2 (2020 onwards), post the onset of the COVID‐19 pandemic (Figure 2B). The study indicated a statistically significant 32.12% reduction in the incidence of children diagnosed with “Disease of the respiratory system” due to COVID‐19 outbreaks and related NPIs (*p* < 0.001).

The top 10 disease diagnoses during these two periods are presented in Table 1. Relative to Period 1, notable decreases in the proportions of inpatients diagnosed with “Pneumonia,” “Disorders of newborns related to short gestation or low birth weight, not elsewhere classified decreased,” “Birth asphyxia,” “Transitory disorders of carbohydrate metabolism specific to fetus or newborn,” and “Disorders of the retina” was observed during Period 2, whereas the opposite trend was evident regarding “Neonatal hyperbilirubinemia,” “Respiratory distress of newborn,” “Hemolytic disease of the fetus or newborn,” “Congenital pneumonia,” and “Structural developmental anomalies of the respiratory system.” The top 10 disease diagnoses among inpatients each year are shown in Table S1.

**Table 1:** Disease distributions among inpatients in Period 1 and Period 2.

Period 1		Period 2	
Pneumonia	25.57%	Neonatal hyperbilirubinemia	21.62%
Disorders of newborn related to short gestation or low birth weight, not elsewhere classified	18.95%	Respiratory distress of newborn	20.58%
Neonatal hyperbilirubinemia	15.19%	Pneumonia	17.46%
Respiratory distress of newborn	12.79%	Congenital pneumonia	7.20%
Birth asphyxia	4.44%	Infections of the fetus or newborn, unspecified	3.14%
Hemolytic disease of fetus or newborn	2.68%	Hemolytic disease of fetus or newborn	3.06%
Congenital pneumonia	2.40%	Disorders of newborn related to short gestation or low birth weight, not elsewhere classified	1.75%
Transitory disorders of carbohydrate metabolism specific to fetus or newborn	1.91%	Structural developmental anomalies of the respiratory system	1.28%
Disorders of the retina	0.65%	Birth asphyxia	0.76%
Structural developmental anomalies of the respiratory system	0.48%	Transitory disorders of carbohydrate metabolism specific to fetus or newborn	0.69%

## 4 DISCUSSION

Ensuring the health and well‐being of children is a priority for national healthcare systems, both in China and globally. The disease spectrum observed among young children has changed markedly in recent years.^15^, ^16^ In this study, the ICD‐11 classification was used to analyze the spectrum of neonatal diseases in China, comparing the disease profiles of neonatal inpatients before and after the COVID‐19 lockdown was lifted.

The COVID‐19 pandemic has highlighted many different health system‐specific vulnerabilities. Following the initial outbreak of the disease, marked declines in patient numbers were observed each year. Parents tended to be more protective of newborns and infants during the pandemic, with most patients affected by minor illnesses relying on remote consultation or self‐care. Many parents and guardians concerned about the risk of infection may have also elected to forgo hospital care during the pandemic. This coincided with a significant decline in pediatric infectious disease cases, with prior evidence supporting a reduction in pediatric viral and bacterial infection rates.^9^ This observation aligns with the understanding that many pathogens, like SARS‐CoV‐2, spread through similar modes of transmission, such as respiratory droplets and close contact.

NPIs are public health measures that seek to control or prevent the transmission of SARS‐CoV‐2 in the community. These measures, including social distancing, travel restrictions, risk communication, and appropriate resource allocation, were the primary tools employed to constrain the global spread of COVID‐19.^17^ Several studies have confirmed the value and efficacy of NPIs as approaches to slowing the progression of the pandemic.^18^

Children tend to be less frequently infected with SARS‐CoV‐2 than adults, and they typically experience fewer symptoms and less severe disease when infected with this virus.^19^ Ongoing research suggests that the innate immune response may be central to the greater resistance of children to SARS‐CoV‐2.^9^, ^20^ Frequent exposure to various pathogens, particularly during the early years of life, has been suggested to improve the efficacy of the innate immune response, leading to a more effectively trained immune system.^21^ Public health measures to contain the COVID‐19 pandemic may have inadvertently led to an immunity gap among newborns and young children, potentially increasing their susceptibility to various infections.^9^, ^22^ A proactive effort must, therefore, be made to address infectious disease outbreaks among children.

## 5 CONCLUSIONS

The neonatal period is defined as the first 4 weeks of life, and it is the time during which children are most vulnerable, with an estimated 2.4 million newborns dying during this period in 2020, according to the WHO. The epidemiological characterization of neonatal diseases may contribute to an improved understanding of the pathophysiological basis for these conditions among professionals, aiding efforts to establish disease‐related risk factors.^23^ These findings will also provide a valuable basis for formulating health policies. Our research suggests that improvements in clinical practice are necessary in the following areas: firstly, emphasizing the early detection and warning of infectious diseases; and secondly, optimizing healthcare resources to facilitate fetal intrauterine therapy and assess risks, which can contribute to reducing the incidence of neonatal diseases.

## 6 LIMITATION

To the best of our knowledge, this is the first study to assess the influence of the COVID‐19 pandemic on the spectrum of neonatal disease. The study has several limitations. Firstly, it is a single‐center, hospital‐based study and not a community‐based study. Second, other factors, such as the increased COVID‐19 and influenza vaccine use, might have decreased infectious disease spread; however, these were not assessed. Third, outpatients were not included, thus cannot fully reflect the changes in the spectrum of diseases.

## AUTHOR CONTRIBUTIONS

All authors have read and approved the final version of the manuscript. The corresponding authors had full access to all of the data in this study and takes complete responsibility for the integrity of the data and the accuracy of the data analysis.

## CONFLICT OF INTEREST STATEMENT

The authors declare no conflict of interest.

## ETHICS STATEMENT

The study was approved by the Ethics Committee of the Seventh Medical Center of the PLA General Hospital approved this study (2023‐33). The studies were conducted in accordance with the local legislation and institutional requirements. Written informed consent for participation was not required from the participants or the participants' legal guardians/next of kin because the retrospective design of the study. All methods of this study were carried out per the ethical standards laid down in the 1964 Declaration of Helsinki and its later amendments or comparable ethical standards.

## TRANSPARENCY STATEMENT

The lead author Siqi Hu, Zhichun Feng affirms that this manuscript is an honest, accurate, and transparent account of the study being reported; that no important aspects of the study have been omitted; and that any discrepancies from the study as planned (and, if relevant, registered) have been explained.

## Supporting information

Supplementary Information

## Data Availability

The data sets supporting the conclusions of this article are included within the article and its additional file.
