# Supplementary material for: The influence of the COVID‐19 pandemic on the spectrum of neonatal disease in a tertiary hospital in China: A retrospective analysis
Source: Health Sci Rep. 2024 Feb 25;7(2):e1928. doi: 10.1002/hsr2.1928 (PMC10895153; doi:10.1002/hsr2.1928)
Supplement: Supplementary file 1 — Supplementary Information [file HSR2-7-e1928-s001.docx]

Additional Table 1. Top ten diseases

| 2018 | | 2019 | | 2020 | | 2021 | | 2022 | |
| --- | --- | --- | --- | --- | --- | --- | --- | --- | --- |
| Pneumonia | 27.39% | Pneumonia | 23.41% | Neonatal hyperbilirubinaemia | 23.30% | Respiratory distress of newborn | 22.45% | Neonatal hyperbilirubinaemia | 20.49% |
| Disorders of newborn related to short gestation or low birth weight, not elsewhere classified | 25.69% | Neonatal hyperbilirubinaemia | 17.60% | Respiratory distress of newborn | 22.81% | Neonatal hyperbilirubinaemia | 20.59% | Pneumonia | 19.69% |
| Neonatal hyperbilirubinaemia | 13.14% | Respiratory distress of newborn | 16.37% | Pneumonia | 15.03% | Pneumonia | 18.56% | Respiratory distress of newborn | 14.36% |
| Respiratory distress of newborn | 9.76% | Disorders of newborn related to short gestation or low birth weight, not elsewhere classified | 10.99% | Congenital pneumonia | 8.00% | Congenital pneumonia | 7.46% | Congenital pneumonia | 5.56% |
| Birth asphyxia | 4.29% | Congenital pneumonia | 5.24% | Infections of the fetus or newborn, unspecified | 4.45% | Haemolytic disease of fetus or newborn | 3.34% | Haemolytic disease of fetus or newborn | 2.90% |
| Haemolytic disease of fetus or newborn | 2.24% | Birth asphyxia | 4.61% | Haemolytic disease of fetus or newborn | 2.91% | Infections of the fetus or newborn, unspecified | 2.72% | Disorders of newborn related to short gestation or low birth weight, not elsewhere classified | 2.81% |
| Transitory disorders of carbohydrate metabolism specific to fetus or newborn | 1.88% | Haemolytic disease of fetus or newborn | 3.21% | Transitory disorders of carbohydrate metabolism specific to fetus or newborn | 1.76% | Disorders of newborn related to short gestation or low birth weight, not elsewhere classified | 2.42% | Structural developmental anomalies of the respiratory system | 2.23% |
| Disorders of the retina | 1.21% | Transitory disorders of carbohydrate metabolism specific to fetus or newborn | 1.94% | Birth asphyxia | 1.48% | Structural developmental anomalies of the respiratory system | 2.00% | Disorders of the retina | 2.09% |
| Vomiting in newborn | 0.72% | Structural developmental anomalies of the respiratory system | 1.04% | Chronic respiratory disease originating in the perinatal period | 1.33% | Sepsis of fetus or newborn | 1.24% | Infections of the fetus or newborn, unspecified | 1.71% |
| Other specified infections of the fetus or newborn | 0.72% | Clinical findings in the digestive system | 1.03% | Neonatal aspiration syndromes | 1.12% | Other specified infections of the fetus or newborn | 1.05% | Birth asphyxia | 0.71% |
